# Klotho Lacks a Vitamin D Independent Physiological Role in Glucose Homeostasis, Bone Turnover, and Steady-State PTH Secretion *In Vivo*

**DOI:** 10.1371/journal.pone.0031376

**Published:** 2012-02-03

**Authors:** René Anour, Olena Andrukhova, Eva Ritter, Ute Zeitz, Reinhold G. Erben

**Affiliations:** Institute of Physiology, Pathophysiology and Biophysics, Department of Biomedical Sciences, University of Veterinary Medicine, Vienna, Austria; University of Crete, Greece

## Abstract

Apart from its function as co-receptor for fibroblast growth factor-23 (FGF23), Klotho is thought to regulate insulin signaling, intracellular oxidative stress, and parathyroid hormone (PTH) secretion in an FGF23 independent fashion. Here, we crossed Klotho deficient (*Kl^−/−^*) mice with vitamin D receptor (VDR) mutant mice to examine further vitamin D independent functions of Klotho. All mice were fed a rescue diet enriched with calcium, phosphorus, and lactose to prevent hyperparathyroidism in VDR mutants, and were killed at 4 weeks of age after double fluorochrome labeling. *Kl^−/−^* mice displayed hypercalcemia, hyperphosphatemia, dwarfism, organ atrophy, azotemia, pulmonary emphysema, and osteomalacia. In addition, glucose and insulin tolerance tests revealed hypoglycemia and profoundly increased peripheral insulin sensitivity in *Kl^−/−^* mice. Compound mutants were normocalcemic and normophosphatemic, did not show premature aging or organ atrophy, and were phenocopies of VDR mutant mice in terms of body weight, bone mineral density, bone metabolism, serum calcium, serum phosphate, serum PTH, gene expression in parathyroid glands, as well as urinary calcium and phosphate excretion. Furthermore, ablation of vitamin D signaling in double mutants completely normalized glucose and insulin tolerance, indicating that Klotho has no vitamin D independent effects on insulin signaling. Histomorphometry of pancreas islets showed similar beta cell volume per body weight in all groups of animals. In conclusion, our findings cast doubt on a physiologically relevant vitamin D and Fgf23 independent function of Klotho in the regulation of glucose metabolism, bone turnover, and steady-state PTH secretion *in vivo*.

## Introduction

The *Klotho* gene was named after the Greek goddess spinning the thread of life. Lack of *Klotho* is known to shorten life span and to cause organ atrophy, vascular calcifications, osteomalacia, hypercalcemia, hyperphosphatemia, elevated circulating vitamin D hormone, and increased peripheral insulin sensitivity in mice [1]–[3]. The major sites of expression of Klotho are the distal renal tubule and the choroid plexus in the brain [1]. The *Klotho* gene encodes for a transmembrane protein, acting as a co-receptor for fibroblast growth factor-23 (FGF23) [4]. The main functions of FGF23 signaling in the kidney are the suppression of vitamin D hormone synthesis, and the suppression of renal tubular phosphate reabsorption [5]. In addition, the extracellular fraction of Klotho can be shed and released into circulation. By a putative sialidase activity, and possibly a weak β-glucuronidase activity, the secreted Klotho protein may have the ability to remove sialic acid or other terminal sugars from sugar chains of multiple membrane glycoproteins, thereby altering their function and membrane abundance. In this context, it has been suggested that the secreted Klotho protein modulates the activity and membrane abundance of the epithelial calcium channel (transient receptor potential cation channel subfamily V member 5, TRPV5) in distal renal tubules [6], [7], the function of the sodium-phosphate transporter-2a (NaPi2a) in proximal renal tubules [8], and the activity of the insulin-like growth factor-1 (IGF-1) receptor [9] in an FGF23 independent fashion. It is thought that circulating Klotho prolongs life span by inhibiting insulin and IGF-I signaling [9], and subsequently lowering intracellular oxidative stress [10]. Moreover, Klotho may also be involved in parathyroid hormone (PTH) secretion through an association with plasma membrane Na^+^-K^+^-ATPase, and a regulatory role in the recruitment of this protein to the plasma membrane in response to hypocalcemic stimuli [11].

Taken together, Klotho may have a dual function. Together with the FGF receptor 1c or other FGF receptor subtypes the transmembrane form of Klotho acts as a co-receptor for FGF23 [4], [12]–[14]. In addition, Klotho *per se* may induce functional changes in transmembrane proteins through its enzymatic activity or its ability to form protein complexes. However, the close association between FGF23/Klotho signaling and vitamin D metabolism has hampered the clear dissection of the FGF23 and vitamin D independent functions of Klotho *in vivo*.

To determine the function of Klotho *per se* in the regulation of glucose, mineral, and bone homeostasis by a genetic approach, we mated Klotho deficient (*Kl^−/−^*) mice with vitamin D receptor mutant mice (VDR^Δ/Δ^) characterized by a non-functioning VDR [15]. Importantly, *Kl^−/−^* mice lack both the transmembrane and the secreted from of Klotho, because both protein isoforms are encoded by the same gene [1], [16]. All mice were kept on a so called “rescue diet” enriched with calcium, phosphorus, and lactose. This elegant dietary tool prevents secondary hyperparathyroidism in VDR mutants [15], [17], [18]. We found that *Kl^−/−^/*VDR^Δ/Δ^ compound mutant mice were phenocopies of VDR^Δ/Δ^ mutants in terms of mineral and bone homeostasis, circulating PTH levels, parathyroid gene expression, as well as glucose and insulin tolerance, suggesting that Klotho lacks a physiologically relevant vitamin D and Fgf23 independent role in the regulation of glucose metabolism, bone turnover, and steady-state PTH secretion *in vivo*.

## Results and Discussion

### Premature aging in *Kl^−/−^* mice is rescued by ablation of VDR signaling

**Figure 1:**
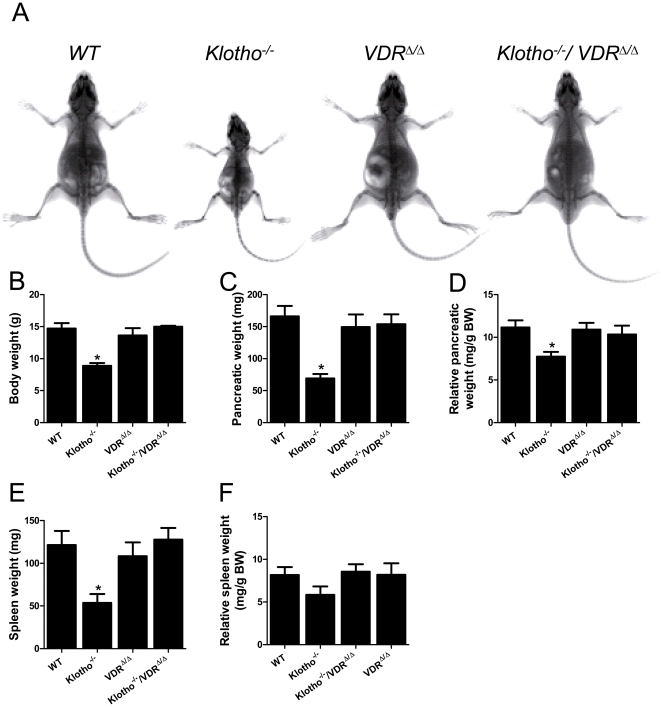
Dwarfism and organ atrophy in *Klotho^−/−^* mice is rescued by ablation of VDR function. (**A**) X-ray image of 4-week-old *WT*, *Kl^−/−^*, VDR^Δ/Δ^, and *Kl^−/−^*/VDR^Δ/Δ^ mice on rescue diet shows dwarfism in *Kl^−/−^* mice. VDR^Δ/Δ^ and compound mutants display normal body size. (**B–F**) Relative to WT controls, *Kl^−/−^* mice show reduced body weight (BW), reduced absolute and relative organ weight of the pancreas, and reduced absolute but not relative organ weight of the spleen. Body weight and organ weights are indistinguishable between VDR^Δ/Δ^ and compound mutants. All animals were fed the rescue diet. Each data point is the mean ± SEM of 4–8 animals per genotype. * denotes P<0.05 vs. WT mice, 1-way ANOVA followed by Student-Newman-Keuls test.

**Figure 2:**
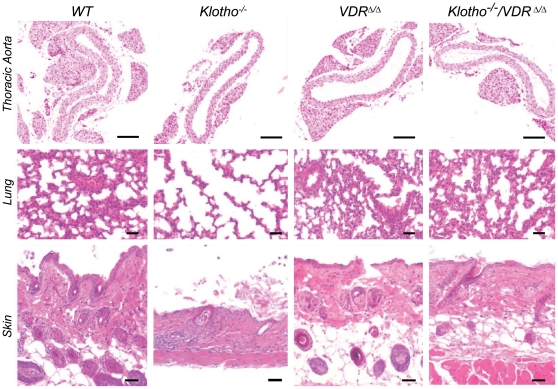
Ablation of VDR signaling rescues skin atrophy and pulmonary emphysema in *Klotho^−/−^* mice. Upper panel shows representative von Kossa-stained sections counterstained with nuclear fast red of thoracic aortae from 4-week-old *WT*, *Kl^−/−^*, VDR^Δ/Δ^, and *Kl^−/−^*/VDR^Δ/Δ^ mice. No ectopic calcifications are present in *Kl^−/−^* mice but a thinning of the aortic wall is evident. Middle and lower panels show HE stained sections. Pulmonary emphysema and severe skin atrophy with almost complete lack of subcutaneous fat tissue is clearly evident in *Kl^−/−^* mice, but absent in *Kl^−/−^*/VDR^Δ/Δ^ compound mutants. All animals were fed the rescue diet. Five-µm-thick paraffin sections. Bar = 200 µm.

In accordance with earlier work from other groups [1], [2], [16], 4-week-old *Kl^−/−^* mice on rescue diet displayed dwarfism (Fig. 1A) which was reflected in a 40% reduction in body weight compared with wild-type (WT) animals (Fig. 1B). Absolute organ weights of spleen and pancreas were significantly lower in *Kl^−/−^* relative to WT mice (Fig. 1C & E). However, when corrected by body weight, true organ atrophy was present only in the pancreas (Fig. 1D), but not in the spleen (Fig. 1F) of *Kl^−/−^* mice. VDR^Δ/Δ^ and *Kl^−/−^/*VDR^Δ/Δ^ compound mutant mice were undistinguishable from their WT littermates in terms of body or organ weight (Fig. 1A–F). Pathohistological findings in *Kl^−/−^* animals included striking atrophy of skin and subcutaneous adipose tissue, pulmonary emphysema, and thinning of the aortic wall, symptoms which were completely absent in VDR^Δ/Δ^ and *Kl^−/−^/*VDR^Δ/Δ^ mice (Fig. 2).

It is well known that *Kl^−/−^* mice develop ectopic calcifications by the age of 6–8 weeks [1], [19]. In our study, ectopic calcifications were absent in von Kossa-stained tissue sections from 4-week-old *Kl^−/−^* mice or from any of the other genotypes (Fig. 2 and data not shown), indicating that these symptoms manifest 2–4 weeks later in life of *Kl^−/−^* mice. All mice in the current study were fed the rescue diet. The lack of ectopic calcifications in 4-week-old *Kl^−/−^* mice suggests that feeding the rescue diet enriched with calcium and phosphorus to *Kl^−/−^* mice does not profoundly aggravate the phenotype, at least not in 4-week-old mice. In accordance with earlier studies [1], [19], we found ectopic calcifications in skin, kidneys, aorta, arteries, and lungs of 8-week-old *Kl^−/−^* but not in WT, VDR^Δ/Δ^ and *Kl^−/−^*/VDR^Δ/Δ^ mice on rescue diet (data not shown).

Another argument in favor of the notion that the major physiological consequence of *Klotho* deficiency is defective Fgf23 signaling is that the phenotypes of *Fgf23^−/−^* and *Kl^−/−^* mice share striking similarities, and that 4-week-old *Fgf23^−/−^* mice can also be completely rescued by ablation of the vitamin D signaling pathway [20], [21].

### *Kl^−/−^*/VDR^Δ/Δ^ compound mice have normal mineral and bone homeostasis

**Figure 3:**
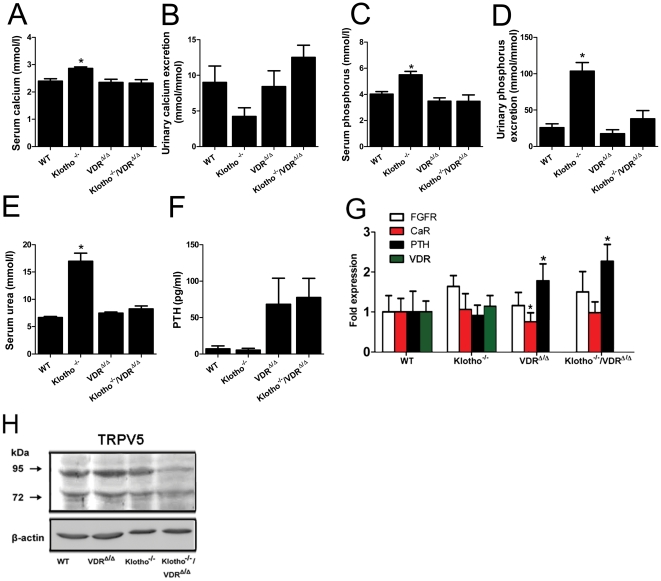
Defects in mineral homeostasis observed in *Klotho^−/−^* mice are normalized in *Klotho^−/−^*/VDR^Δ/Δ^ double mutant mice. (**A–D**) Analysis of mineral homeostasis in serum and urine shows hypercalcemia, hyperphosphatemia, and hyperphosphaturia in *Kl^−/−^* mice compared with WT controls, whereas compound mutants are normocalcemic and normophosphatemic. (**E**) *Kl^−/−^* mice display about 2- to 3-fold higher serum urea values than WT mice. (**F**) Serum intact parathyroid hormone (PTH) tends to be higher in VDR and compound mutants, relative to WT mice (n = 8–10 each). (**G**) mRNA abundance assessed in isolated parathyroid glands by qRT-PCR reveals higher PTH mRNA abundance in VDR and compound mutants relative to WT mice. Relative expression of Fgf receptor 1c (FGFR) and PTH is not different between VDR^Δ/Δ^ and compound mutants, but also not different between WT and *Kl^−/−^* mice. Calcium-sensing receptor (CaR) mRNA expression was lower in VDR^Δ/Δ^ compared with WT mice. Using primers located in the deleted region of the VDR in gene-targeted VDR^Δ/Δ^ mice, relative VDR mRNA expression was not different between WT and *Kl^−/−^* mice, and undetectable in VDR^Δ/Δ^ and compound mutants. (**H**) Representative Western blotting image of core (75 kD) and complex (95 kD) glycosylated TRPV5 protein expression in renal cortical total membrane fractions of 4-week-old WT, VDR^Δ/Δ^, *Kl^−/−^* and *Kl^−/−^/*VDR^Δ/Δ^ mice. Membrane abundance of fully glycosylated TRPV5 is lower in *Kl^−/−^* and especially in *Kl^−/−^*/VDR^Δ/Δ^ mice, relative to WT and VDR^Δ/Δ^ mice. All animals were fed the rescue diet. Each data point is the mean ± SEM of 4–10 animals per genotype. * denotes P<0.05 vs. WT mice, 1-way ANOVA followed by Student-Newman-Keuls test.

*Kl^−/−^* mice were hypercalcemic and hyperphosphatemic (Fig. 3A & C). Renal calcium excretion in *Kl^−/−^* mice tended to be lower, but was not significantly different from WT controls (Fig. 3B). Serum urea was increased in *Kl^−/−^* mice (Fig. 3E), which might reflect altered hepatic nitrogen metabolism or a beginning impairment in renal function. Serum creatinine tended to non-significantly elevated in *Kl^−/−^* mice relative to WT controls (data not shown). *Klotho* deficiency is known to be associated with increased tubular reabsorption of phosphate by an upregulation of proximal tubular apical membrane expression of NaPi-2a [8]. However, renal phosphate excretion was actually increased in *Kl^−/−^* relative to WT mice (Fig. 3D). It is likely that the increased tubular reabsorption of phosphate in *Kl^−/−^* mice is overridden by the profoundly increased filtered phosphate load caused by severe hyperphosphatemia.

Compound mutants lacking both *Klotho* and a functional VDR were normocalcemic and normophosphatemic, and did not show changes in serum PTH levels or renal calcium and phosphate excretion relative to VDR^Δ/Δ^ mice (Fig. 3A–F). Although statistically not significant, there was a trend for moderately increased serum PTH levels in VDR^Δ/Δ^ and *Kl^−/−^*/VDR^Δ/Δ^ mutants compared with WT and *Kl^−/−^* mice (Fig. 3F). We made a very similar observation in an earlier study in which the rescue diet also did not fully normalize serum PTH in 4-week-old VDR^Δ/Δ^ and *Fgf23^−/−^*/VDR^Δ/Δ^ mice [21]. It is possible that the rescue diet used in this and our earlier study (20% lactose, 2.0% Ca, 1.25% P) may not be able to fully correct serum PTH levels in very young VDR mutant mice. In line with the trend for increased circulating intact PTH, we found significantly increased PTH mRNA abundance in isolated parathyroid glands from VDR and compound mutants, relative to WT mice (Fig. 3G). However, there were no differences in parathyroid mRNA abundance of the Fgf receptor 1c, calcium-sensing receptor, and PTH genes between WT and *Kl^−/−^* mice, and also not between VDR and compound mutants (Fig. 3G). However, mRNA expression of the calcium-sensing receptor was slightly, but significantly, lower in VDR^Δ/Δ^ mice as compared to WT controls. Using primers located in the deleted region of the VDR (corresponding to the deleted exon 2), VDR mRNA expression was not different between WT and *Kl^−/−^* mice, and was undetectable in VDR^Δ/Δ^ mice and compound mutants.

Collectively, our data suggest that absence of transmembrane and secreted Klotho does not influence steady-state PTH secretion or PTH gene transcription *in vivo*. In contrast, it has been reported earlier that isolated parathyroid glands from *Kl^−/−^* mice secrete less PTH in response to a hypocalcemic stimulus in organ culture compared with parathyroid glands from WT mice [11]. A possible explanation for this discrepancy may be preexistent changes in parathyroid chief cells due to lifelong hypercalcemia in *Kl^−/−^* mice, which may alter PTH secretion in organ culture. Alternatively, acute regulation of PTH secretion induced by changes in extracellular Ca^2+^ concentrations *in vitro* may be a more sensitive system to examine the molecular role of certain molecules in PTH secretion, compared with the steady-state conditions in the current experiment. Under steady-state conditions, a possible essential role of Klotho in PTH secretion may be counterbalanced by yet unknown mechanisms in parathyroid chief cells.

In our experiment, we did not find significant differences in renal calcium excretion between the genotypes. Work from other groups has shown that the plasma membrane expression of TRPV5 is downregulated in the distal renal tubule of *Klotho* deficient mice, leading to decreased renal tubular reabsorption of calcium [6], [22]. TRPV5-mediated apical calcium entry is the rate-limiting step in distal tubular transcellular calcium transport [23]. In accordance with these earlier reports, we also found reduced membrane abundance of distal tubular TRPV5 in *Kl^−/−^* and *Kl^−/−^*/VDR mice, relative to WT and VDR mutant mice (Fig. 3H). These changes should result in increased renal loss of calcium in *Kl^−/−^* and *Kl^−/−^*/VDR mice, which was not observed. The absence of *Klotho* deficiency-induced alterations in renal calcium handling in spite of the presence of altered membrane expression of TRPV5 in our study might be explained by the rapid skeletal growth, the very high bone turnover, and the high concentration of urinary calcium in 4-week-old mice on rescue diet, potentially masking more subtle differences in renal calcium handling. All groups showed urinary calcium concentrations in the range of 5–10 mmol/mmol creatinine, which is very high. In addition, because PTH increases the expression and activates the TRPV5 channel [23], [24], the slightly higher PTH levels in VDR and compound mutants might also contribute to masking the urinary calcium leak in compound mutants. The trend for reduced renal calcium excretion in *Kl^−/−^* mice can probably be explained by hypervitaminosis D which is known to be present in these mice [3]. The vitamin D hormone is an important regulator of renal calcium reabsorption [15], [23].

**Figure 4:**
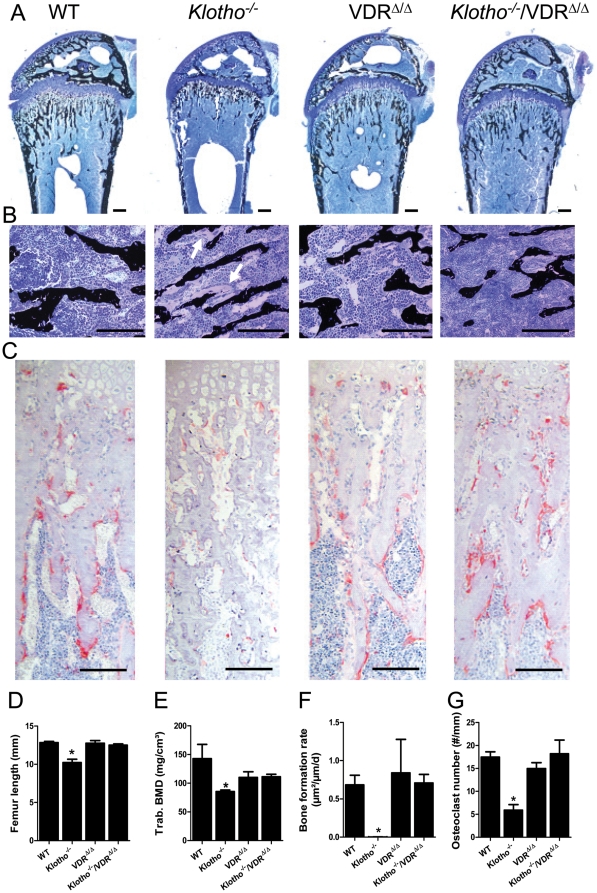
Bone phenotype of *Klotho^−/−^* mice is rescued in *Klotho^−/−^/VDR^Δ/Δ^* compound mutants. (**A–B**) Three-µm-thick undecalcified plastic sections of distal femurs of 4-week-old WT, *Kl^−/−^*, VDR^Δ/Δ^, and *Kl^−/−^*/VDR^Δ/Δ^ compound mutant mice, stained with von Kossa/McNeal. Low power view (A) shows normal bone architecture in VDR mutants and *Kl^−/−^*/VDR^Δ/Δ^ compound mutants, while narrowing and premature ossification of the growth plate area is evident in *Kl^−/−^* mice. High power view (B) reveals severe osteomalacia (thickened osteoid seams are indicated by arrows) in cancellous bone of *Kl^−/−^* mice, but normal mineralization in the other genotypes. (**C**) Undecalcified sections of distal femurs histochemically stained for tartrate resistant acid phosphatase (TRACP) activity show normal numbers of osteoclasts in VDR^Δ/Δ^ and *Kl^−/−^*/VDR^Δ/Δ^ compound mutants, but profoundly reduced osteoclast numbers in the distal metaphysis of *Kl^−/−^* mice. Bar in A–C = 200 µm. (**D**) Femur length measured by calipers is lower in *Kl^−/−^* mice, but remains unchanged in VDR and compound mutants, relative to WT controls. (**E**) Trabecular volumetric bone mineral density (BMD) of the distal femoral metaphysis measured by peripheral quantitative computed tomography (pQCT). VDR mutants and *Kl^−/−^*/VDR^Δ/Δ^ compound mutants show a slightly decreased femoral trabecular BMD, relative to WT mice. (**F–G**) Measurement of cancellous bone formation rate (F) and osteoclast number (G) in the distal femoral metaphysis by histomorphometry reveals almost undetectable calcein-based bone formation and decreased osteoclast numbers in *Kl^−/−^* mice, but normal bone turnover in VDR and compound mutant mice. All animals were fed the rescue diet. Each data point is the mean ± SEM of 6–7 animals per genotype. * denotes P<0.05 vs. WT mice, 1-way ANOVA followed by Student-Newman-Keuls test.

The bone phenotype of *Kl^−/−^* mice was characterized by a narrowed growth plate (Fig. 4A), severe osteomalacia (Fig. 4B), reduced femur length (Fig. 4D), reduced volumetric trabecular bone mineral density (BMD, Fig. 4E), very low cancellous bone formation rate (Fig. 4F), and reduced osteoclast numbers (Fig. 4C & G). The trend for lower femoral metaphyseal BMD in VDR and compound mutants compared with WT mice (Fig. 4E) can very likely be explained by the slightly increased serum PTH levels found in both groups of mice (Fig. 3F). However, bone histology, femoral volumetric BMD, bone formation rate, and osteoclast numbers were indistinguishable in VDR^Δ/Δ^ and *Kl^−/−^*/VDR^Δ/Δ^ double mutants, indicating that Klotho is dispensable for normal bone metabolism in VDR-ablated mice.

### *Klotho* deficiency lacks a vitamin D independent regulatory effect on glucose homeostasis *in vivo*

**Figure 5:**
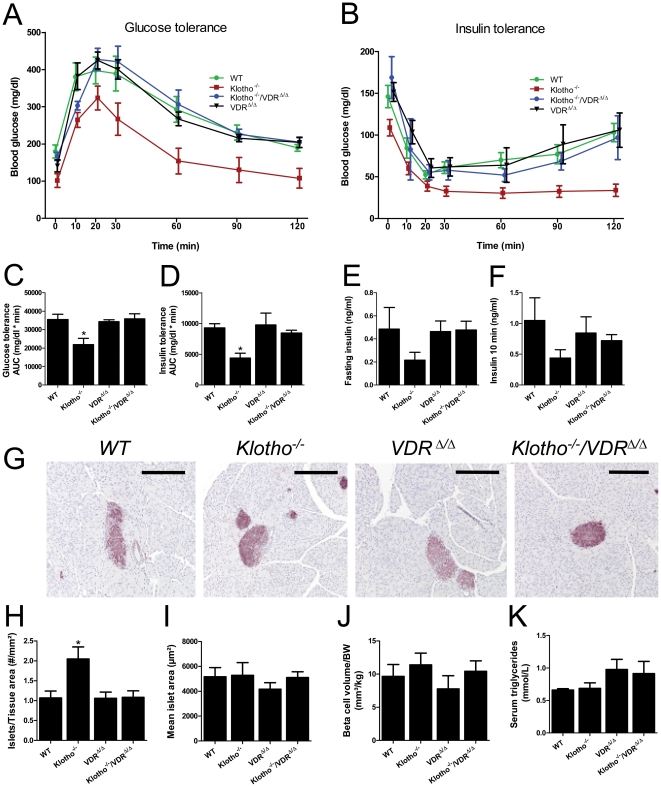
Normalization of increased insulin sensitivity in *Klotho^−/−^* mice by ablation of vitamin D signaling. (**A–D**) Glucose tolerance and insulin tolerance tests in 4-week-old *WT*, *Kl^−/−^*, VDR^Δ/Δ^, and *Kl^−/−^/VDR* ^Δ/Δ^ mice show hypoglycemia and profoundly improved glucose tolerance (A, C) and enhanced insulin sensitivity (B, D) in *Kl^−/−^* mice. Ablation of vitamin D signaling in compound mutants completely normalizes glucose and insulin tolerance. The area under the curve (AUC) for glucose and insulin tolerance tests is shown in C and D. Glucose (1.5 mg/g body weight) was administered subcutaneously at time 0 after two hours of fasting. Insulin (0.6 IE/kg) was injected intraperitoneally at time 0. (**E–F**) Fasting insulin and insulin serum levels measured 10 min after a subcutaneous glucose challenge (1.5 mg/g) are not significantly different between the groups, but tend to be lower in *Kl^−/−^* mice. (**G–J**) Histomorphometric analysis of anti-insulin-stained pancreatic paraffin sections (G) shows higher number of islets per tissue area in *Kl^−/−^* compared with WT mice (H), but unchanged mean islet size (I) and beta cell volume per kg BW (J) in all groups of animals. Bar in G = 200 µm. (**K**) Serum triglycerides are similar in all groups of animals. All animals were fed the rescue diet. Each data point is the mean ± SEM of 6–8 animals per genotype. * denotes P<0.05 vs. WT mice, 1-way ANOVA followed by Student-Newman-Keuls test.

Consistent with earlier reports [1], [2], we found hypoglycemia (Fig. 5A & B), improved subcutaneous glucose tolerance (Fig. 5A & C), and profoundly augmented insulin sensitivity (Fig. 5B & D) in *Kl^−/−^* mice. Histomorphometry of pancreas islets showed an increased number of islets of Langerhans per pancreas tissue area, but unchanged size of individual islets in *Kl^−/−^* relative to WT mice (Fig. 5G–I). However, total beta cell volume per kg body weight was normal in *Kl^−/−^* mice (Fig. 5J), showing that *Kl^−/−^* mice are characterized by atrophy of the exocrine but not of the endocrine pancreas. Ablation of vitamin D signaling in double mutants completely normalized glucose and insulin tolerance (Fig. 5A–D), indicating that Klotho has no vitamin D independent effects on insulin sensitivity. Morphology of pancreatic islets and beta cell volume remained unchanged in VDR and *Kl^−/−^*/VDR^Δ/Δ^ compound mutants relative to WT mice (Fig. 5G–J). Previous studies suggested that serum triglycerides can be used to monitor hepatic insulin sensitivity under steady-state conditions in mice [25]. Serum triglycerides did not differ between any of the groups (Fig. 5K), suggesting that *Klotho* deficiency was not associated with altered hepatic insulin sensitivity.

Taken together, our data show that neither the secreted nor the transmembrane form of Klotho is involved in the physiological regulation of glucose homeostasis in mice. In agreement with our study, two rat models of insulin resistance failed to detect a regulation of Klotho, and administration of insulin sensitizers to rats did not increase renal Klotho expression [26]. However, several open questions remain. It is currently unclear why *Klotho* and *Fgf23* deficient mice show such a pronounced increase in insulin sensitivity, why the life span of *Klotho* transgenic mice is 20–30% higher compared to WT mice [9], and why single nucleotide polymorphisms of the human *KLOTHO* gene are associated with life span, stroke and early onset coronary artery disease [27]–[29]. Previously, it was thought that circulating Klotho induces insulin resistance and prolongs life by suppressing insulin signaling [9]. Decreased insulin signaling increases life span in several species from worms and flies to mice, and appears to be a highly conserved mechanism to prolong life (reviewed in [30]). It is clear that we cannot rule out a pharmacological effect of Klotho on insulin signaling. However, our data unequivocally show that Klotho does not have a vitamin D independent physiological function in the regulation of peripheral insulin sensitivity. Thus, in light of our data we hypothesize that the observed associations between *KLOTHO* gene polymorphisms and aging or age-related diseases in humans, as well as the extended life span in Klotho transgenic mice, are not due to altered insulin signaling, but due to subtle long-term functional alterations in FGF23 signaling, thereby altering vitamin D and phosphate metabolism. In accordance with this hypothesis, a 13-year-old girl with a homozygous missense mutation in human *KLOTHO* showed severe tumoral calcinosis, but no signs of accelerated aging [31]. It is presently unclear whether the increased insulin sensitivity in *Fgf23^−/−^* and *Kl^−/−^* mice is caused by hyperphosphatemia, hypercalcemia, increased circulating levels of the vitamin D hormone, or simply by the runting phenotype. It has been suggested that phosphate toxicity may be an important mediator of the increased insulin sensitivity in *Fgf23^−/−^* and *Kl^−/−^* mice [32]. However, unequivocal experimental evidence for this hypothesis is lacking. Clearly, more experimentation is necessary to find out the molecular mechanism behind the changes in glucose homeostasis seen in *Fgf23^−/−^* and *Kl^−/−^* mice.

In conclusion, our data cast doubt on the hypothesis that transmembrane or secreted Klotho has physiologically relevant, vitamin D independent, functions in the regulation of carbohydrate metabolism, bone turnover, and steady-state PTH secretion. Rather, the major physiological function of Klotho appears to be its function as co-receptor for Fgf23.

## Materials and Methods

### Animals

All animal studies were approved by the Ethical Committee of the University of Veterinary Medicine, Vienna and by the Austrian Federal Ministry of Science and Research and were undertaken in strict accordance with prevailing guidelines for animal care and welfare (permit number 68.205/0083-II/10b/2008). All efforts were made to minimize suffering. Wild-type (WT), *Kl^−/−^*, VDR^Δ/Δ^, and *Kl^−/−^*/VDR^Δ/Δ^ mice were generated by intercrossing VDR^+/Δ^ [15] and *Kl^+/−^* (Lexicon Genetics, Mutant Mouse Regional Resource Centers, University of California, Davis) double heterozygous animals. VDR mutant mice were genotyped by routine PCR as described [15]. Genotyping of *Klotho* mutants was performed with the following primers (f, forward; r, reverse): SelC2f 5′TGCCTGCTTGCCGAATATCA3′, KlothoUni2 5′TGGAATGCCTTGGTGCTATC3′, and KlothoWT2f 5′GTCTTCGGCCTTGTTCTACCA3′. All animals were fed a rescue diet (Ssniff, Soest, Germany) which contained 2.0% calcium, 1.25% phosphorus, 20% lactose and 600 IU vitamin D/kg, starting from 16 days of age. The mice were kept at 24°C with a 12 hour/12 hour light/dark cycle, and were allowed free access to the rescue diet and tap water. Spontaneous urine was collected before necropsy. At necropsy, the mice were killed by exsanguination from the abdominal V. cava under anesthesia with ketamine/xylazine. Serum samples were taken to assess mineral homeostasis.

### Organ histology and pancreatic islet histomorphometry

Organs were fixed in 4% paraformaldehyde overnight, and were processed for paraffin histology and haematoxylin/eosin (HE) or von Kossa staining by routine methods. The paraffin-infiltrated pancreas of each animal was cut in ∼2 mm thick slices perpendicular to the long axis of the organ using a razor blade. All slices from each animal were placed into one embedding cassette with the cut surface facing downwards. Subsequently, the paraffin blocks were sectioned at 5 µm thickness. With the use of this approach, each section contained tissue from all pancreas regions. For immunohistochemical detection of insulin-expressing cells in paraffin sections of the pancreas, sections were deparaffinized, incubated for 15 min in 3% hydrogen peroxide in PBS to block endogenous peroxidase activity, and, after blocking with 20% rabbit serum, incubated for 2 h at room temperature with guinea pig anti-porcine insulin antiserum diluted 1∶2,000 (DAKO, Hamburg, Germany). Bound antibody was detected with peroxidase-conjugated rabbit anti-guinea pig IgG (DAKO). Vector VIP (Vector) was used as enzyme substrate. Finally, the sections were counterstained with Mayer's hematoxylin. Histomorphometry of pancreatic islets was performed on anti-insulin stained sections as described in detail previously [33].

### Biological chemistry

Serum calcium, phosphorus, creatinine, urea, triglycerides, and cholesterol, as well as urinary calcium, phosphorus, and creatinine were analyzed on a Hitachi 912 Automatic Analyzer (Roche). PTH serum concentrations were assessed using a two-sided enzyme-linked immunosorbent assay specific for intact mouse PTH (Immutopics).

### Bone analysis

Femur length was measured with calipers. BMD of the left femora was assessed by peripheral quantitative computed tomography (QCT) using an XCT Research M+ peripheral QCT machine (Stratec Medizintechnik), using a voxel size of 0.070 mm. Distal femoral metaphyseal BMD was calculated as mean over two slices located 2.0 and 2.5 mm proximal from the articular surface. For assessment of dynamic histomorphometric indices, the mice were injected with calcein (20 mg/kg) 2 days and 1 day before sacrifice. Processing of bone specimens and cancellous bone histomorphometry in the distal femoral metaphysis was performed as described earlier [34], [35].

### Glucose and insulin tolerance tests

For glucose tolerance tests, the mice were kept individually on hunger grids with free access to tap water for a period of 2 h before testing. Subsequently, 1.5 mg glucose per g body weight was administered subcutaneously. Blood glucose levels were measured with a standard test system (One Touch Ultra, Lifescan) at time 0, 10, 20, 30, 60, 90 and 120 min, using 2-µl whole blood samples obtained by scarifying a tail vein. For insulin tolerance tests, mice were kept individually with free access to tap water and were deprived of food at time 0. Subsequently, the mice were intraperitoneally injected with 0.6 IU insulin (Humalog, Insulin lispro, Lilly) per kg body weight. Blood glucose levels were monitored as described above.

### RNA isolation and quantitative RT-PCR

Intact microdissected parathyroid glands were isolated under a stereo microscope, using a modification of the methods described by Imura et al. [11] and Rodriguez et al [36]. In brief, mice were anesthetized with ketamine/xylazine and transcardially perfused with DMEM/F12 (Sigma) for 10 min before thyro-parathyroidectomy. Thyroid and parathyroid glands isolated together with the trachea were placed in RNAse-free centrifugal tubes (Millipore), and were washed twice in DMEM/F12. Subsequently, parathyroid glands were dissected free of the thyroid gland based on their opaque appearance under a stereo microscope within 2 min. The isolated parathyroid glands were immediately lyzed in guanidinium thiocyanate buffer, and homogenized by passing the suspension stepwise through 21G–27G needles. Total RNA was extracted with phenol/chloroform, precipitated using isopropanol, and then treated with RQ1 RNase-free DNase (Promega). After first-strand cDNA synthesis (iScript cDNA Synthesis Kit, Bio-Rad), quantitative RT-PCR was performed on a Rotor-Gene™ 6000 (Corbett Life Science) using QuantiFast™ SYBR Green PCR Kit (Qiagen). Ct values of target genes were normalized to *hprt* gene abundance. Regulation of gene expression was calculated using the approach of the efficiency corrected quantification model according to Pfaffl [37].

### Kidney total cell membrane isolation

Mouse kidney cortices were homogenized in a homogenizing buffer [20 mM Tris (pH 7.4/HCl), 5 mM MgCl_2_, 5 mM NaH_2_PO_4_, 1 mM ethylenediamine tetraacetic acid (pH 8.0/NaOH), 80 mM sucrose, 1 mM phenyl-methylsulfonyl fluoride, 10 µg/ml leupeptin and 10 µg/ml pepstatin], and subsequently centrifuged for 15 min at 4,000 *g*. Supernatants were transferred to a new tube and centrifuged for an additional 30 min at 16,000 *g*.

### Western Blot

Total cell membrane samples were solubilized in Laemmli sample buffer, fractionated on SDS-PAGE (30 µg/well) and transferred to a nitrocellulose membrane (Thermo Scientific). Immunoblots were incubated overnight at 4°C with primary polyclonal including anti-TRPV5 antibodies (1∶3,000, Lifespan Biosciences) and monoclonal mouse anti-β-actin (1∶5,000, Sigma) in 2% (w/v) bovine serum albumin (BSA, Sigma) in a TBS-T buffer [150 mM NaCl, 10 mM Tris (pH 7.4/HCl), 0.2% (v/v) Tween-20]. After washing, membranes were incubated with horseradish peroxidase-conjugated secondary antibodies (Amersham Life Sciences). Specific signal was visualized by ECL kit (Amersham Life Sciences). The protein bands were quantified by Image Quant 5.0 software (Molecular Dynamics). The expression levels were normalized to Ponceau S stain.

### Statistical analyses

Statistics were computed using SPSS for Windows 17.0 (SPSS Inc.). The data were analyzed by one way analysis of variance (ANOVA) followed by Student-Newman-Keuls multiple comparison test. P values of less than 0.05 were considered significant.
